# Administration of Metabiotics Extracted From Probiotic *Lactobacillus rhamnosus* MD 14 Inhibit Experimental Colorectal Carcinogenesis by Targeting Wnt/β-Catenin Pathway

**DOI:** 10.3389/fonc.2020.00746

**Published:** 2020-06-02

**Authors:** Mridul Sharma, Geeta Shukla

**Affiliations:** Department of Microbiology, Panjab University, Chandigarh, India

**Keywords:** metabiotics, bioactive substances, probiotics, colon cancer, aberrant crypt foci

## Abstract

**Background and Objective:** The cellular microenvironment, diet, and lifestyle play a key role in the occurrence of colorectal cancer. Due to its rising trend, attempts are being made to devise novel biointerventions as adjunct to conventional therapies to prevent this deadly disease. “Metabiotics,” the beneficial metabolic signatures of probiotics are emerging as potential anticancer agent due to their ability to alter metabolic processes in the gut lumen and reduce the severity of colon carcinogenesis. Although beneficial attributes of metabiotics have been elucidated *in vitro*, yet their anticancer mechanism *in vivo* needs to be explored. Thus, the present study was performed to envisage anticancer potential of metabiotic extract obtained from indigenous probiotic, *Lactobacillus rhamnosus* MD 14, in early experimental colon carcinogenesis.

**Materials and Methods:** Sprague–Dawley rats were daily administered with low, medium, and high dose of metabiotic extract orally along with a single dose of weekly intraperitoneal injection of 1,2-dimethylhydrazine up to 6 weeks and monitored for the markers of early colon carcinogenesis.

**Results:** It was observed that the medium dose of metabiotic extract attenuated early colon carcinogenesis by reducing fecal procarcinogenic enzymes, oxidants, aberrant crypt foci, vis-à-vis downregulating oncogenes [K-ras, β-catenin, Cox-2, nuclear factor kappa B (NF-κB)] and upregulating tumor suppressor p53 gene leading to almost normal colon histology.

**Conclusions:** It can be suggested that metabiotics modulate experimental colorectal cancer and could be used as a promising alternative of probiotics, particularly in immunocompromised individuals.

## Introduction

Colorectal cancer (CRC) remains one of the deadliest cancers with different molecular phenotypes, resistance to chemotherapies, recurrence after surgery, and high mortality rate (1, 2). Pathogenesis of colorectal cancer has been extensively investigated, and there is enough evidence to reveal that genetic mutations accompanied with epigenetic alterations and defective immunological signaling pathways are the chief contributors, which are generally triggered by imbalanced intestinal microbiome. Thus, regaining the balance of intestinal microbiota via oral supplementation of beneficial microbes, the probiotics, is gaining importance. However, it has been observed that introducing a foreign live strain, even a probiotic, could impose various adverse effects such as bacteremia, septicemia, infective endocarditis, abdominal abscesses, and allergic sensitization particularly in highly sensitive individuals such as infants, elderly, immunocompromised, and genetically predisposed individuals (3–5).

The functional metabolites secreted by probiotics, designated as “metabiotics” provide advantage of being safer and probably more effective strategy either as prophylactic or therapeutic agent (6). Metabiotics refer to “the structural components of probiotic microorganisms and/or their metabolites and/or signaling molecules with a determined chemical structure that can optimize host-specific physiological functions, regulatory, metabolic and/or behavior reactions connected with the activity of host indigenous microbiota” (7). Various metabolites produced by probiotic organisms include organic acids, glycoproteins, peptides, exopolysaccharides, polyphosphates, etc. Short-chain fatty acids (SCFAs) such as acetate, propionate, and butyrate constitute the major metabiotics shown to participate in immune regulation along with maintenance of normal physiology of the gut (8).

Owing to the presence of various bioactive components, metabiotics have been shown to exhibit antineoplastic and antioxidant potential leading to effective physiological and biochemical modulation, which may attenuate the precancerous lesions in the large intestine (9). Additionally, an unbalanced gut microbiome as observed in CRC, may encourage the production of bacterial enzymes such as β-glucosidase, β-glucuronidase, azoreductase, and nitroreductase, the agents responsible for generation of carcinogens as well as toxic metabolites including secondary bile salts, aromatic amines, and reactive oxygen species (10, 11). A slight decline in fecal pH may inactivate enzymatic activity of the harmful bacteria such as *Escherichia coli*, clostridia, fusobacteria, and their binding to the surrounding epithelium (12). Metabiotics, mainly SCFAs, have been found to induce acidosis and promote apoptosis in CRC cells, thereby reducing the incidence and severity of colonic neoplasm (13, 14). Furthermore, antioxidant capacity associated with probiotic metabolites such as conjugated linoleic acids may prevent tumor formation by scavenging free radicals and inhibiting peroxidation (15–17). Potentially anticarcinogenic metabiotics repress colonic neoplastic growth mainly by accelerating apoptosis and inhibiting the proliferation of cancer cells (18). Moreover, in CRC, the interplay of oncogenes through various molecular pathways such as Wnt signaling/Janus kinase (JAK)-signal transducer and activator of transcription (STAT)/transforming growth factor beta (TGF-β) pathway leads to inflammation, aberrant crypt foci, oxidative stress, and the formation of tumor and its uncontrolled division (19). Davis et al. and Yu et al. have reported that the gut microbial bioactive substances play a key role in targeting multiple steps of these metabolic pathways (20, 21).

Recently, we observed that metabiotics obtained from indigenous probiotic, *Lactobacillus rhamnosus* MD 14 (accession no. MH656799), showed potent antigenotoxic and cytotoxic potential against Caco-2 and HT-29 colon cancer cells (22). Since information pertaining to the *in vivo* efficacy of metabiotics with anticancerous potential is not available, it was pertinent to assess the molecular antitumorigenic potential of metabiotics extracted from *L. rhamnosus* MD 14 in early colon cancer induced by 1,2-dimethylhydrazine (DMH) in Sprague–Dawley rats.

## Methods

### Chemicals

All the chemicals used in the present study were of analytical grade and obtained from standard companies. DMH and TRIzol reagent were procured from Sigma Chemical Company, St. Louis, MN, USA. Primers were obtained from Eurofins Pvt. Ltd, Bangalore, India. De Man, Rogosa, and Sharpe (MRS) broth, MRS agar, ethyl acetate, carboxymethyl cellulose (CMC), Hank's balanced salt solution (HBSS), trypan blue, sodium chloride, potassium chloride, sodium dihydrogen phosphate, potassium dihydrogen phosphate, trisodium citrate and tris-hydrochloric acid, ethylenediaminetetraacetic acid (EDTA), tris-saturated phenol, agarose, ethidium bromide, and acridine orange were purchased from Hi-Media Pvt. Ltd Laboratories Mumbai, India.

### Animals

Male Sprague–Dawley (SD) rats (100–200 g) were procured from the inbred population of Central Animal House, Panjab University, Chandigarh, India. Rats were kept in standard polypropylene cages (three animals per cage) with a wire mesh top and a hygienic bed of husk (regularly changed) in room with 12-h light/dark cycle, constant temperature (24°C), and humidity. These animals were acclimatized for 1 week and provided with normal standard pellet diet, water *ad libitum*. Care and usage of animals were as per the principles and guidelines of the Ethics Committee (PU/45/99/CPCSEA/IAEC/2017/26) of the Animal Care of Panjab University, Chandigarh, India, till the end of the experimental period.

### Study Design

#### Induction of Colon Carcinogenesis

Chemical carcinogen, DMH, was dissolved in 1 mM EDTA saline, and pH was adjusted to 7.0 with 1 mM NaOH. A single dose of DMH (20 mg/kg body weight) was given intraperitoneally (i.p.), once in a week to animals, and the treatment was continued for 6 weeks (23).

#### Probiotic Culture

Probiotic, *L rhamnosus* MD 14 isolated in our research laboratory from infant feces, was found to possess antigenotoxic and anticancer properties. Furthermore, it was identified using 16S rRNA sequencing and deposited in the National Center for Biotechnology Information (NCBI) database, GenBank with sequential accession number, MH656799 (22). The probiotic was grown in MRS medium having pH 6.5, incubated in aerobic conditions at 37°C for 24 h, and was maintained by regular subculturing on MRS agar at an interval of 15 days.

#### Metabiotic Extraction and Dose Preparation

Cell-free supernatant (CFS) was prepared by adjusting the optical density of 24-h-old probiotic culture to 0.8 corresponding to 10^8^-10^9^ CFU/ml, followed by cold centrifugation at 4,000 g for 10 min and filtered through a 0.2-μm membrane filter. Organic extract was prepared from CFS of the probiotic strain. Briefly, CFS (200 ml) was mixed vigorously with equal volume of ethyl acetate for 15 min in a separatory funnel and was kept as such at 37°C until the formation of two separate layers. The concentrated upper layer was collected, and extraction procedure was repeated thrice. Finally, the organic concentrate was pooled, dried, and dissolved in carboxy methyl cellulose at a concentration of 65 mg/ml optimized by *in vitro* experiments (22). Different doses were prepared in 0.5% CMC used as the solvent and were administered via orogastric gavage to the animals.

#### Identification of Active Components in Metabiotic Extract

In order to identify the presence of various components in the effective metabiotic extract, liquid chromatography mass spectrometry was performed in the central instrumentation facility of Panjab University, Chandigarh, India using Waters Micromass Q-T of LCMS Instrument, Unisol YVR C18 column, electrospray positive ionization with 20 μl injection volume, 0.8 ml/min flowrate, acetonitrile with 0.1% formic acid as mobile phase, nitrogen (6–7 bar) and argon (5–6 bar) gases employing 300°C desolvation temperature, 110°C source temperature, and 3,000 V capillary voltage.

### Experimental Design

**Figure 1:**
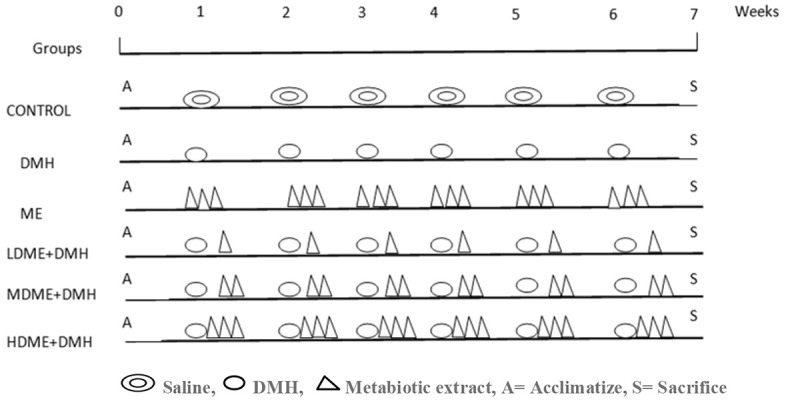
Schematic representation of experimental design for the induction of colon carcinogenesis in Sprague–Dawley (SD) rats by 1,2-dimethylhydrazine (DMH).

Thirty-six animals were randomly divided into six groups (Figure 1) and were treated as follows.

- Group I: Control
  Six animals were administered with EDTA saline, once a week i.p. and 0.5% CMC daily, orally, via orogastric gavage, for 6 weeks.
- Group II: 1,2-dimethylhydrazine (DMH)
  Six animals received DMH (20 mg/kg) in 1 mM EDTA saline i.p. once a week for 6 weeks.
- Group III: Metabiotic extract (ME)
  Six animals were administered daily with a single dose of metabiotic extract (4 ml/kg) orally, for 6 weeks.
- Group IV: Lowest dose (1 ml/kg) of metabiotic extract (LDME + DMH)
- Six animals received daily a single dose of metabiotic extract (1 ml/kg) orally, for 6 weeks.
- Group V: Medium dose (2 ml/kg) of metabiotic extract (MDME + DMH)
  Six animals received daily a single dose of metabiotic extract (2 ml/kg) orally, for 6 weeks.
- Group VI: Highest dose (4 ml/kg) of metabiotic extract (HDME + DMH)
- Six animals received daily a single dose of metabiotic extract (4 ml/kg) orally, for 6 weeks.

However, a single dose of DMH was given i.p. once a week for 6 weeks along with daily oral feeding of respective doses of metabiotic extract to animals belonging to groups IV, V, and VI.

### Follow-Up of the Animals

After respective treatment of animals belonging to different groups, body mass, growth rate, fecal pH, lactobacilli count, and procarcinogenic enzymes (nitroreductase, β-glucosidase assay, β-glucuronidase) were estimated. However, 1 week after the completion of respective treatment for 6 weeks, animals were sacrificed by injecting ketamine hydrochloride (80 mg/kg) intraperitoneally followed by cervical dislocation. Blood was drawn by cardiac puncture, and serum was separated to assess lipid profile (total cholesterol, total lipids) and liver function tests, i.e., serum bilirubin, alanine transaminase (ALT), aspartate transaminase (AST), and alkaline phosphatase (ALP). Whole colon was removed to assess the macroscopic lesions, aberrant crypt foci (ACF), apoptosis, oxidant malondialdehyde (MDA), antioxidants [superoxide dismutase (SOD), reduced glutathione (GSH), glutathione peroxidase (Gpx)] levels, and histological alterations. In order to analyze, the expression of proto-oncogenes [Cox-2, β-catenin, nuclear factor kappa B (NF-κB), K-ras] and tumor suppressor gene (p53), colonic tumors were taken, processed immediately for RNA isolation followed by complementary DNA (cDNA) preparation and analyzed by real-time quantitative PCR (qPCR).

### Estimation of Body Mass, Growth Rate, Fecal Lactobacilli Count, and pH

Body mass of rats belonging to all the groups was recorded weekly on an ordinary balance (SD-300, S.D Fine Chemicals Ltd, Chandigarh, India). Growth rate was calculated as the difference between the final weight and the initial weight divided by the total number of days (24). In order to assess the effect of supplementation of metabiotics on beneficial bacteria particularly lactobacilli in the colon, freshly voided fecal material (0.5 g/rat) from each group once in a week was homogenized in normal saline, serially diluted, and plated on MRS agar. The plates were incubated at 37°C for 24 h, and CFU were recorded (24). To monitor fecal pH, freshly passed feces were collected from each rat once per week up to 6 weeks, emulsified with neutral 0.9% sodium chloride, and was recorded with Deluxe model 101 E pH meter (25).

### Fecal Enzyme Analysis

Before sacrifice, each animal was placed in separate cage for collecting fresh fecal samples. Briefly, for β-glucuronidase and β-glucosidase assay, fecal samples were suspended in cold prereduced potassium phosphate buffer (0.1 M, pH 7.0) and in Tris–HCl buffer (0.2 M, pH 7.8) for nitroreductase assay. The fecal suspension was homogenized, sonicated for 3 min at 4°C, cold centrifuged at 500 g for 15 min. The enzymes were assayed immediately from the supernatant as per Verma and Shukla, and protein concentration was also determined (26, 27). Fecal nitroreductase activity was expressed as microgram of m-aminobenzoic acid formed per hour per milligram of fecal protein. The β-glucosidase activity was expressed as microgram of nitrophenol formed per hour per milligram of fecal protein. The β-glucuronidase activity was expressed as microgram of phenolphthalein formed per minute per milligram of fecal protein.

### Biochemical Analysis

Blood was collected from each rat after sacrifice by cardiac puncture, and serum was used to assess the biochemical parameters like lipid profile including total cholesterol, total lipids, and liver function tests including serum bilirubin, ALT, AST, and alkaline phosphatase (ALP) by autoanalyzer (Sysnex XP-100).

### Examination of Colon and Determination of Apoptosis

After cervical dislocation of animals, the colon of each animal was removed, cleansed, and observed macroscopically for the presence of lesions and inflammation. For ACF examination, distal colon was cut into small sections of 2–5 cm, stained with 0.2% methylene blue, and the total number of ACF was counted using a light microscope (23). To analyze apoptosis, the entire colon was flushed with Ca^2+^- and Mg^2+^-free phosphate-buffered saline (PBS), cut longitudinally to expose the lumen. The cut colon was placed in warm Ca^2+^- and Mg^2+^-free HBSS, 30 mM EDTA, 5 mM dithiothreitol, and 0.1% bovine serum albumin, incubated at 37°C on a shaker for 15 min, and mucosal side was gently scraped to isolate colonocytes. The isolated colonocytes were stained with ethidium bromide/acridine orange (EtBr/AO) and observed under a fluorescence microscope (28).

### Assessment of Oxidant and Antioxidant Level

After sacrificing the animals, the colon was taken and colonic tissue homogenates were prepared in 0.15 M PBS (pH 7.2) using a potter Elvehjem homogenizer. For the preparation of postmitochondrial supernatant (PMS), tissues homogenates were centrifuged at 16,000 g for 10 min at 4°C, and supernatant was labeled as PMS. Protein concentration in colonic tissue homogenate and PMS was measured as per Lowry et al. (27). The amount of MDA formed, a measure of lipid peroxidation, was assayed in colonic tissue homogenate according to the method of Wills (29). The results were expressed as nanomoles of MDA per milligram of protein. Superoxide dismutase (SOD) activity was assayed in the PMS of tissue homogenate according to the method of Kono (30) and was expressed as units of SOD per milligram of protein, where one unit activity is defined as the amount of SOD required to inhibit the rate of reduction in nitroblue tetrazolium (NBT) by 50%. GSH levels were estimated in tissue homogenates as per Ellman (31), absorbance was measured by taking OD at 412 nm, and results were expressed as micromoles of GSH/milligram of protein. The GPx activity was estimated in PMS as per the method of Wendel, (32) and was defined as units of GPx per milligram of protein where one unit is expressed as the amount of enzyme that will catalyze the oxidation of 1.0 μM reduced glutathione to oxidized glutathione per minute at 25°C by H_2_O_2_ at pH 7.0.

### Effect of Metabiotic Administration on Gene Expression

The effect of supplementation of metabiotic extract on the expression of various oncogenes, i.e., Cox-2, β-catenin, NF-κB, K-ras, and tumor suppressor gene p53 involved in early colorectal carcinogenesis was quantified by real-time qPCR. After sacrificing, the colon of each animal was removed and cleansed, and total RNA was extracted from colonic mucosa bearing lesions or showing inflammation using the TRIzol reagent (Sigma Aldrich, USA) following manufacturer's protocol. Briefly, cDNA was synthesized from equal amount of total RNA isolated from affected tissue of colon using commercially available kit (Biorad iscript kit 1708891). Real-time PCR was performed in a reaction volume of 20 μl, according to the manufacturer's instructions for the Biorad qPCR kit (iTaq Universal SYBR Green Supermix). Nucleotide primer sequences of rat origin were used (Supplementary Table 1). Briefly, 18.2 μl of Master Mix, 0.8 μl of primer assay (10 × ), and 1 μl of template cDNA (20 ng) were added to each well. After a brief centrifugation, the PCR plate was subjected to 40 cycles of the following conditions: (i) PCR activation at 95°C for 20 s, (ii) denaturation at 95°C for 3 s, and (iii) annealing at 60/59/52/60/55/56°C for β-actin, Cox-2, β-catenin, NF-κB, K-ras, and p53, respectively, for 20 s. All samples and controls were run in triplicate on a Mastercycler realplex system (Biorad CFX 96). The quantitative data were analyzed using a comparative threshold method (2^−ΔΔCq^), and the fold inductions of the samples were compared with the control sample. β-actin was used as an internal reference gene to normalize the expression of the target genes.

### Histological Observation

The colonic segments were fixed immediately in 10% buffered formalin, processed, stained with hematoxylin and eosin, and examined for histological alterations by a light microscope.

### Statistical Analysis

Results were expressed as mean ± standard deviation. The intergroup variation was assessed by one-way analysis of variance (ANOVA) followed by Dunnett/Tukey's/251 Bonferroni post hoc test. *P* < 0.05 was considered as significant. Graph pad PRISM-5.0 software 252 was used for analysis.

## Results

### Active Components in the Metabiotic Extract

**Figure 2:**
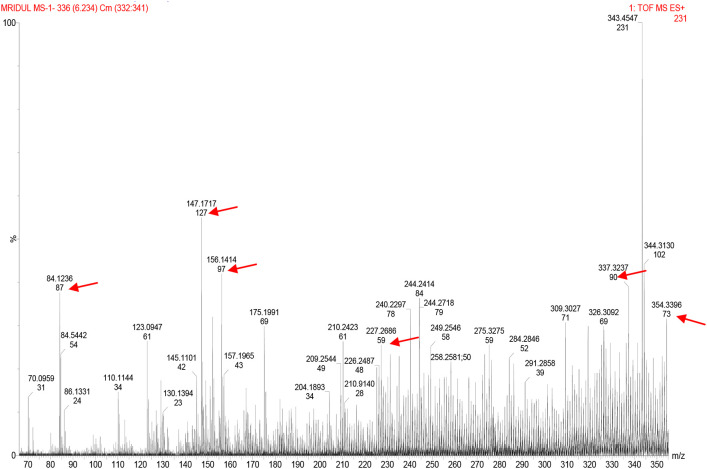
Liquid chromatography mass spectrometry chromatogram of metabiotic extract depicting the presence of various chemical compounds as indicated by arrows: acetate (mol. wt, 59), butyrate (mol. wt, 87), propionate (mol. wt, 73), acetamide (mol. wt, 127), thiocyanic acid (mol. wt, 97), and oxalic acid (mol. wt, 90).

LCMS peaks of the metabiotic extract depicted the presence of various components such as short chain fatty acids, i.e., acetate, butyrate, propionate, as well as other active compounds, i.e., acetamide, thiocyanic acid, and oxalic acid in the metabiotic extract (Figure 2).

### Increased Growth Rate, Fecal Lactobacilli Count, and Lower pH

The body mass of animals belonging to all groups increased at each point of observation, but increase in body mass was variable (Supplementary Figures 1A,B). Furthermore, it was observed that all animals receiving metabiotic extract had increased growth rate despite DMH treatment. In particular, the growth rate was significantly (*p* < 0.05) more in MDME + DMH animals than DMH-treated animals and respective counter controls (ME, LDME + DMH, HDME + DMH). Interestingly, the count of beneficial lactobacilli, an indicator of healthy gut microbiome, increased in feces significantly (*p* < 0.05) in all the metabiotic-administered animals, despite DMH treatment (ME, LDME + DMH, MDME + DMH, HDME + DMH) compared with DMH-treated group of animals (Supplementary Figure 1C). Notably, feces of the animals administered with DMH had alkaline pH in the range of 8.0–8.8 at each point of observation during the experimental period. More specifically, it was found that fecal pH of the animals treated with ME showed a shift from alkaline to acidic pH by fourth week, and fecal pH was significantly (*p* < 0.05) lower in MDME + DMH-treated animals followed by HDME + DMH in comparison to DMH-treated animals (Supplementary Figure 1D).

### Reduced Fecal Enzymes

**Figure 3:**
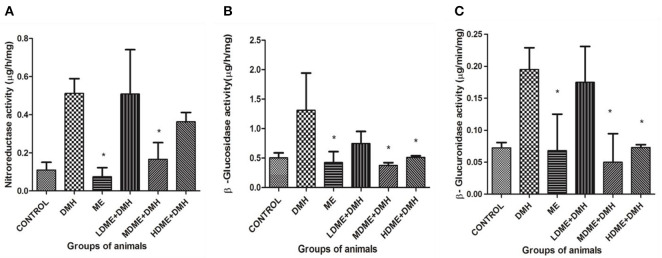
Fecal enzymes activity in different groups of animals: **(A)** nitroreductase, **(B)** β-glucosidase, and **(C)** β-glucuronidase. Experiment was performed in triplicates. Values are expressed as mean ± SD. *p < 0.05 vs. 1,2-dimethylhydrazine (DMH)-treated.

It was found that activity of fecal enzymes, i.e., nitroreductase, β-glucuronidase, and β-glucosidase, decreased in animals administered with metabiotic extract but significant (*p* < 0.05) reduction was recorded in MDME + DMH animals followed by HDME + DMH, LDME + DMH, respectively, in comparison to DMH-treated group of animals (Figure 3).

### Modulation of Biochemical Parameters

It was observed that both lipid profile (serum cholesterol, total lipids) and liver function tests (bilirubin, ALT, AST, ALP) in all animals administered with metabiotic extract (ME, LDME + DMH, MDME + DMH, HDME + DMH) were similar to that of control animals but was significantly (*p* < 0.05) less in comparison to DMH-treated group of animals (Table 1).

**Table 1:** Serum lipid profile and liver function test of animals belonging to various groups.

**Groups**	**Total cholesterol (mg/dl)**	**Total lipids (mg/dl)**	**Bilirubin (mg/dl)**	**ALT (U)**	**AST (U)**	**Alkaline phosphatase (U)**
Control	77.97 ± 3.9	255.20 ± 2.6	0.89 ± 0.02	235 ± 7.16	64.75 ± 4.3	146.22 ± 3.4
DMH	97.52 ± 5.2	276.47 ± 2.6	1.62 ± 0.04	324.5 ± 5.3	146.52 ± 2.9	225.45 ± 3.6
ME	77.32 ± 4.6^*^	258.77 ± 4.4^*^	0.85 ± 0.03^*^	229.5 ± 7.5^*^	73.32 ± 4.3^*^	145 ± 2.9^*^
LDME + DMH	91.75 ± 7.3	268.97 ± 6.8	0.93 ± 0.03	233 ± 12.3	74.52 ± 4.4	154.25 ± 3.3
MDME + DMH	81.22 ± 7.8^*^	259.3 ± 2.8^*^	0.88 ± 0.07^*^	225.25 ± 4.2^*^	71.55 ± 2.7^*^	154.22 ± 2.3^*^
HDME + DMH	81.51 ± 2.9^*^	262.55 ± 3.5^*^	0.86 ± 0.05^*^	232 ± 6.6^*^	62.52 ± 2.4^*^	155.5 ± 3.1^*^

*Values are expressed as mean ± SD*.

* *p < 0.05 vs. 1,2-dimethylhydrazine (DMH)-treated*.

### Reduced Colonic Lesions, Inflammation, Aberrant Crypt Foci, and Increased Apoptotic Cells

**Figure 4:**
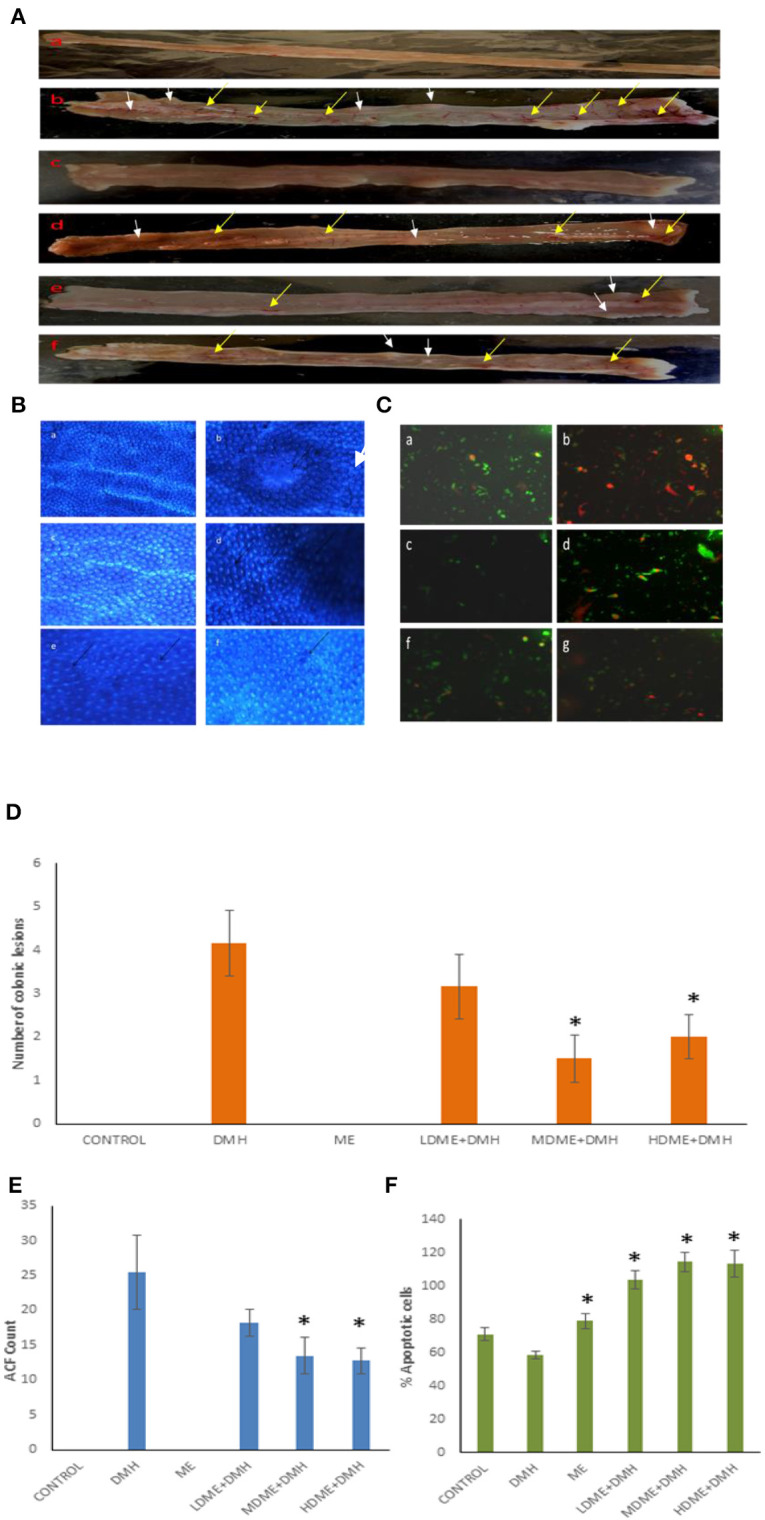
**(A)** Macroscopic observation of inflammation (yellow arrows) and lesions (white arrows). **(B)** Topographic view of the colon stained with methylene blue showing: (a) normal crypts, (b) flat mucin depleted aberrant crypt foci (arrow), (c) almost normal crypts, (d) numerous aberrant crypts (arrows), and (e, f) fewer aberrant crypts. **(C)** Ethidium bromide/acridine orange stained colonocytes of animals belonging to different groups: (a) control, (b) 1,2-dimethylhydrazine (DMH)-treated, (c) metabiotic extract (ME), (d) LDME + DMH, (e) MDME + DMH, (f) HDME + DMH (400 × ). Quantification of **(D)** number of colonic lesions. **(E)** Aberrant crypt foci (ACF) count. **(F)** Percent apoptotic cells in various groups of animals.

The macroscopic examination of the colon revealed the protective potential of metabiotic extract as animals belonging to all metabiotic administered groups (LDME + DMH, MDME + DMH, HDME + DMH) had fewer nodular lesions and decreased inflamed areas compared with increased number of swollen, hard lesions along with thinning of the colon in DMH-treated animals (Figures 4A,D). Microscopically, it was found that the animals belonging to metabiotic-administered groups had significantly (*p* < 0.05) lower ACF counts per colon, i.e., 18.25 ± 1.92 (LDME + DMH), 13.50 ± 2.59 (MDME + DMH), and 12.75 ± 1.78 (HDME + DMH), respectively, in comparison to DMH-treated animals, which showed increased ACF count (25.5 ± 5.25) with flat and mucin-depleted foci (Figure 4B). More specifically, 28.43, 47.05, and 50% reduction in ACF was seen in animals belonging to LDME + DMH-, MDME + DMH-, and HDME + DMH-treated groups, respectively, in comparison to DMH-treated animals (Figure 4E). It was also found that colonocytes isolated from MDME + DMH-treated animals had significantly (*p* < 0.05) higher percentage of apoptotic cells (114% ± 5.85), followed by HDME + DMH (113% ± 8.32) and LDME + DMH (103% ± 5.50) compared with DMH-treated (58% ± 2.08) animals (Figures 4C,F).

### Suppressed Oxidant and Enhanced Antioxidant Level

**Figure 5:**
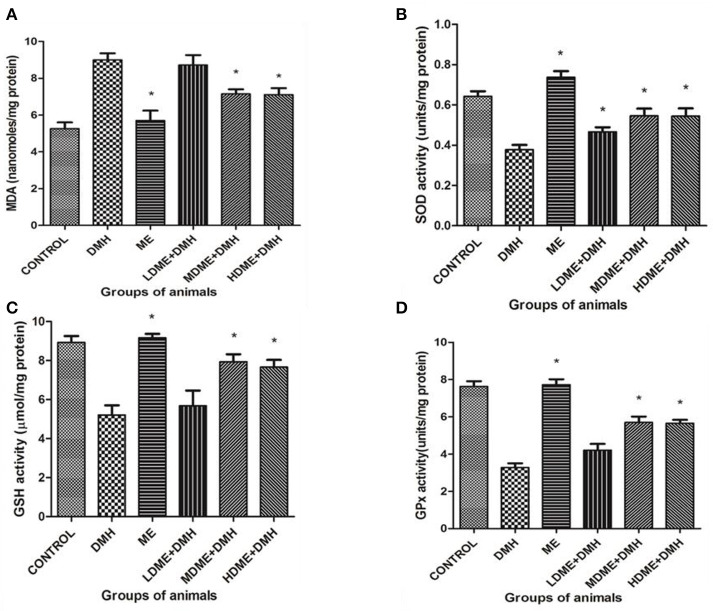
Oxidant and antioxidant levels in animals belonging to different groups: **(A)** malondialdehyde (MDA), **(B)** superoxide dismutase (SOD), **(C)** reduced glutathione (GSH), and **(D)** glutathione peroxidase (Gpx) levels. Experiment was performed in triplicates. Values are expressed as mean ± SD, *p < 0.05 vs. 1,2-dimethylhydrazine (DMH)-treated.

The oral administration of ME significantly (*p* < 0.05) reduced oxidant MDA level and enhanced antioxidant (SOD, GSH, GPx) levels in animals of MDME + DMH and HDME + DMH groups followed by LDME + DMH in comparison to DMH-treated group of animals (Figure 5).

### Modulated Quantitative Gene Expression (Cox-2, β-catenin, NF-κB, K-ras, p53)

**Figure 6:**
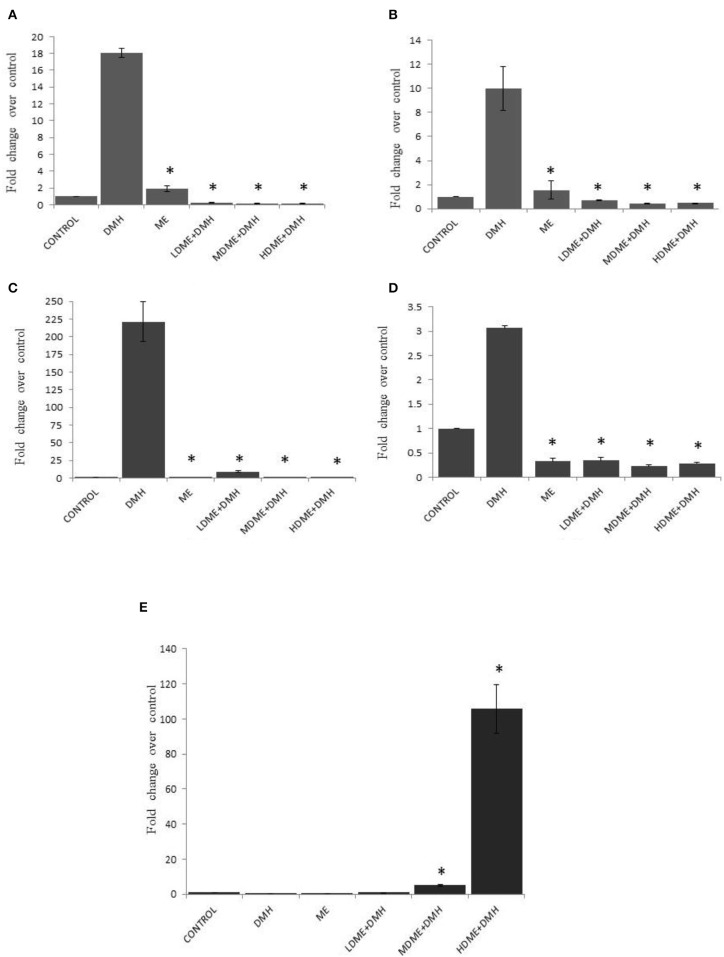
Fold change in expression of molecular markers by real-time PCR in various groups of animals: **(A)** Cox-2, **(B)** β-catenin, **(C)** nuclear factor kappa B (NF-κB), **(D)** K-ras, and **(E)** p53. Experiment was performed in triplicates. Values are expressed as mean ± SD, *p < 0.05 vs. 1,2-dimethylhydrazine (DMH)-treated.

It was found that daily oral administration of metabiotic extract to DMH-treated animals led to significant (*p* < 0.05) downregulation of Cox-2 expression, the proinflammatory marker; β-catenin, the central signaling molecule; NF-κB, the inflammatory mediator; and K-ras, the proto-oncogene particularly in animals belonging to MDME + DMH and HDME + DMH followed by LDME + DMH in comparison to DMH-treated animals. However, p53, a tumor suppressor gene, was upregulated both in HDME + DMH and MDME + DMH animals in comparison to DMH-treated group of animals (Figure 6, Supplementary Table 2). More specifically, the oral administration of metabiotic extract given in the medium dose was most effective in downregulating the expression of oncogenes in comparison to DMH-treated animals.

### Histological Modulation

**Figure 7:**
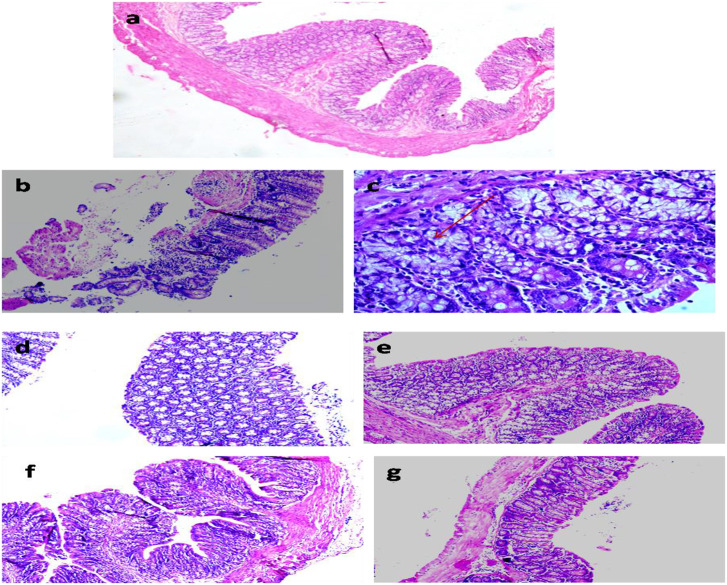
Photomicrograph of the colon of animals belonging to different groups showing **(a)** normal histoarchitecture in control; **(b,c)** severely damaged mucosa and aberrant crypt foci (ACF) (arrow) in 1,2-dimethylhydrazine (DMH)-treated; **(d)** normal colon cells in metabiotic extract (ME); **(e)** colitis in LDME + DMH-treated; **(f,g)** mild inflammation in MDME + DMH and HDME + DMH-treated (H&E staining, 100 × ).

The colon segments of animals belonging to the control showed normal and intact mucosal epithelium (Figure 7a) compared with focal colitis in the form of excess lymphocytes between the glands, hyperplasia, and hyperchromatic nuclei, increased mitotic figures, and presence of ACF indicating preneoplastic changes in the colon (Figures 7b,c). The colon of animals administered with ME showed normal histology similar to control (Figure 7d), but animals belonging to LDME + DMH showed colitis, the focal collection of lymphocytes in the colonic mucosa (Figure 7e). However, supplementation of higher doses of ME restored the colon to almost normal histoarchitecture with inflammatory cells comparable to control in animals belonging to MDME + DMH and HDME + DMH animals (Figures 7f,g).

## Discussion

It is speculated that the gut microbes synthesize, detect, as well as respond to various active metabiotics that are structurally and functionally diverse, offering a wide range of physiological benefits to the host (2, 6). In our earlier *in vitro* study, it was observed that the metabiotic extract of *L. rhamnosus* MD 14 had genotoxicity against DMH and cytotoxicity against Caco-2 and HT-29 colon cancer cells *in vitro* (22). Therefore, it was pertinent to study the *in vivo* anticancerous molecular mechanism of the metabiotic extract in experimental colon carcinogenesis.

The anticancer potential of metabiotics observed in the present study could be a cumulative effect of various bioactive substances present in the metabiotic extract such as acetate, butyrate, propionate, acetamide, thiocyanic acid, and oxalic acid and is in concordance with earlier observations (33, 34). These studies have indicated that SCFAs, mainly butyrate, acetate, and propionate, are the major contributors toward the antitumorigenic potential of metabiotics employing human colon cancer cell lines. However, the role of other bioactive compounds may not be ruled out such as acetamide having anticancerous effect against human cancer cells along with anti-inflammatory and analgesic *in vivo* potentials (35). Similarly, thiocyanic acid and oxalic acid, the other bioactive compounds, have been reported to exhibit antioxidant, antimutagenic, and anticancer activities (36, 37).

The increased growth rate in animals administered either with medium or high doses of metabiotic extract for 6 weeks despite DMH treatment is indicative of better physiology, which could be due to organic acids present in the metabiotic extract that are known to modulate metabolism in animals and have been linked with weight gain (38). In our previous studies too, it was observed that prior oral supplementation of either prebiotic or standard probiotic to DMH-treated rats led to enhanced growth rate in early colon carcinogenesis (23, 26).

The increased lactobacilli count in the feces of all metabiotic-treated animals could be due to improved gut microenvironment that modified the gut microbiota either by modifying gut pH or chemical components in the gut such as SCFAs, lactic acid, and amino acids, which may attenuate colon cancer by replacing harmful bacteria (39, 40). The acidic shift in fecal pH with the oral administration of metabiotic extract could be attributed to the chemical nature of metabiotic extract containing mainly organic acids and is in concordance with previous observations (41, 42). Further, Samelson et al. have suggested that acidic environment may inactivate the harmful bacterial enzymes (β-glucosidase, β-glucuronidase and nitroreductase), which in turn may lead to decrease in the tumor promoting 7-dehydroxylation activity of bile salts (43).

It was observed that either medium or high dose of metabiotic extract modulated the lipid profile of animals by reducing serum cholesterol and total lipids. This may be due to the metabiotic extract containing SCFAs that are known to reduce the level of lipids in blood mainly by inhibiting hepatic cholesterol synthesis and redirection of plasma cholesterol to the liver (44, 45). These scientists have observed that animals administered either with *L. acidophilus* or *Bifidobacterium* had reduced cholesterol levels. Recently, Wang et al. have investigated the relation between cholesterol and colon cancer and found that animals receiving a high amount of cholesterol had stimulated intestinal stem cells that divide at higher rate and enabling tumors to grow extremely faster (46). The liver biomarkers were also regulated in all metabiotic-treated animals as metabiotics are known to regulate endotoxin translocation to the liver by maintaining the gut permeability and stimulating the release of anti-inflammatory cytokines by Kupffer cells (47–49). These scientists have reported that supplementation of probiotics, *L. bulgaricus* and *Streptococcus thermophilus*, to individuals with nonalcoholic fatty liver disease decreased the serum levels of AST and ALT after 3 months of intervention and provided significant protection against liver damage.

It was also noted that oral administration of either medium or high dose of metabiotic extract to animals reduced the inflammation and colonic lesions, which may be attributed to mucosal immune response by various soluble metabolites in the metabiotic extract through activation of immune cells leading to the production of anti-inflammatory cytokines having antitumorigenic potential and is in agreement with our previous studies (23, 26). The decreased aberrant crypt foci in animals followed by oral administration of medium or high dose of metabiotic extract might be due to increased production of mucin, the protective protein by colonic cells that may reduce the number of toxic compounds prevailing in gut microenvironment leading to reduced inflammation and controlled transformation of colonocytes to abnormal cells (50, 51). Furthermore, it has been reported that SCFAs mainly butyrate are the key player in reducing ACF and modulating colonic cell growth, metabolism, and differentiation along with colonic pH (52).

The induction of apoptosis in cancer cells by metabiotic extract may be attributed to altered expression of various proapoptotic genes by butyrate, as it has been reported to promote apoptosis in transformed colonic cells by inhibiting histone deacetylase, thereby affecting the genes involved in cell growth, differentiation, and cell cycle arrest in cancer cells (53–55). Cousin et al. have also observed that metabolites produced by *Propionibacterium freudenreichii* led to apoptosis as evidenced by chromatin condensation, arrest of cell cycle, formation of apoptotic bodies, and caspase activation in human gastric cancer cells (56).

The enhanced levels of antioxidants and decreased oxidants in animals administered with metabiotic extract may be due to the quenching effect of metabiotics, thereby protecting colon cells from toxicity and inflammation and is in accordance with earlier studies (57, 58). Recently, Singh et al. have reported that exopolysaccharides obtained from *L. plantarum* C88 exerted antioxidant effect mainly by scavenging of reactive oxygen species and enhancing both enzymatic or nonenzymatic antioxidant activity along with decreased lipid peroxidation (9). Furthermore, the anticancer potential of metabiotic extract was validated both by macroscopic and histological examination of the colon tissue where animals supplemented either with medium or high dose of metabiotics had fewer lesions, reduced inflammation, and least dysplastic effects with higher number of goblet cells despite DMH treatment. Similarly, earlier studies carried out in our laboratory also showed the prophylactic potential of probiotics by reducing dysplasia and edema and increasing lymphocyte clusters and goblet cells in colonic submucosa of rats in DMH-treated early colon carcinogenesis (24, 28).

On the molecular basis, it was observed that proto-oncogenes (Cox-2, β-catenin, NF-κB, and K-ras) were downregulated while p53; the tumor suppressor gene was upregulated with the administration of metabiotic extract either in medium or high dose to DMH-treated animals. The observed modulation could either be due to inhibition of enzyme histone deacetylase by SCFAs that may have demethylated the promoters of oncogenes or via modulation of Wnt signaling evident by suppression of β-catenin, which in turn may have inhibited the transcription of various oncogenes including Cox-2. The marked inhibition of NF-κB would have resulted into enhanced anti-inflammatory effect and increased expression of its target p53 tumor suppressor gene, which might have repaired the damaged DNA, thereby regaining normal cellular functions. Earlier studies have also reported the downregulation of Cox-2, β-catenin, and NF-κB by probiotic metabolite, butyrate on SW620 and HCT-116 colon cancer cells (59–61). Ronka et al. and Bordonaro et al. have also reported that butyrate and propionate downregulated Cox-2 expression and modulated Wnt signaling in different colon cancer cells (62, 63). Similarly, Guo et al. have found that metabolites of probiotics, *Bifidobacterium infantis* and *L. acidophilus*, downregulated NF-κB expression by modulating hundreds of genes responsible for regulation of immune response, cell division, and apoptosis (64). In our earlier study, too, the prior administration of either probiotic alone or synbiotic have led to the modulation of K*-*ras and p53 in experimental colon carcinogenesis (28, 53).

Taken together, it can be stated that, although both medium and high dose of the metabiotic extract possessed anticancer potential, medium dose was found to be optimum as it effectively attenuated early colon cancer markers and may be economical from production and application point of view. The observed protection offered by metabiotic extract against DMH-induced carcinogenesis might be due to the modification of gut microenvironment mainly due to the presence of various short chain fatty acids (acetate, butyrate, propionate) and other active compounds (acetamide, thiocyanic acid, oxalic acid). These bioactive substances may have led to inactivation of procarcinogenic enzymes, immunomodulation, and increased antioxidant levels that reduced DNA damage by reactive oxygen species. Furthermore, reduced level of proto-oncogenes may have led to induction of apoptosis in abnormally dividing cells, thereby decreased tumorigenesis subsequently affecting various biochemical and signaling pathways involved in colon cancer leading to improved colon cellular morphology.

## Conclusion

In a nutshell, the present study revealed the *in vivo* antitumorigenic potential and modulatory molecular mechanism of metabiotic extract derived from probiotic *L. rhamnosus* MD 14 that may be explored as a novel biointervention in preventing colon cancer particularly in individuals sensitive to live bacteriotherapy, but needs to be validated both by long-term experimental as well as clinical study.

## Data Availability Statement

All datasets generated for this study are included in the article/Supplementary Material.

## Ethics Statement

The animal study was reviewed and approved by Ethics Committee of the Animal Care of Panjab University, Chandigarh, India.

## Author Contributions

MS conducted the research work and drafted the manuscript. GS supervised the study and edited the manuscript.

## Conflict of Interest

The authors declare that the research was conducted in the absence of any commercial or financial relationships that could be construed as a potential conflict of interest.
