# Supplementary material for: Administration of Metabiotics Extracted From Probiotic Lactobacillus rhamnosus MD 14 Inhibit Experimental Colorectal Carcinogenesis by Targeting Wnt/β-Catenin Pathway
Source: Front Oncol. 2020 Jun 2;10:746. doi: 10.3389/fonc.2020.00746 (PMC7326139; doi:10.3389/fonc.2020.00746)
Supplement: Supplementary file 1 [file Data_Sheet_1.pdf]

frontiers

Supplementary Material

Supplementary Figure 1: Effect of different doses of metabiotic extract on animals belonging to different groups on a) body mass; b) growth rate; c) fecal lactobacilli count; d) fecal pH. with respect to weeks post treatment. Values are Mean ± SD, *p < 0.05 versus DMH-treated

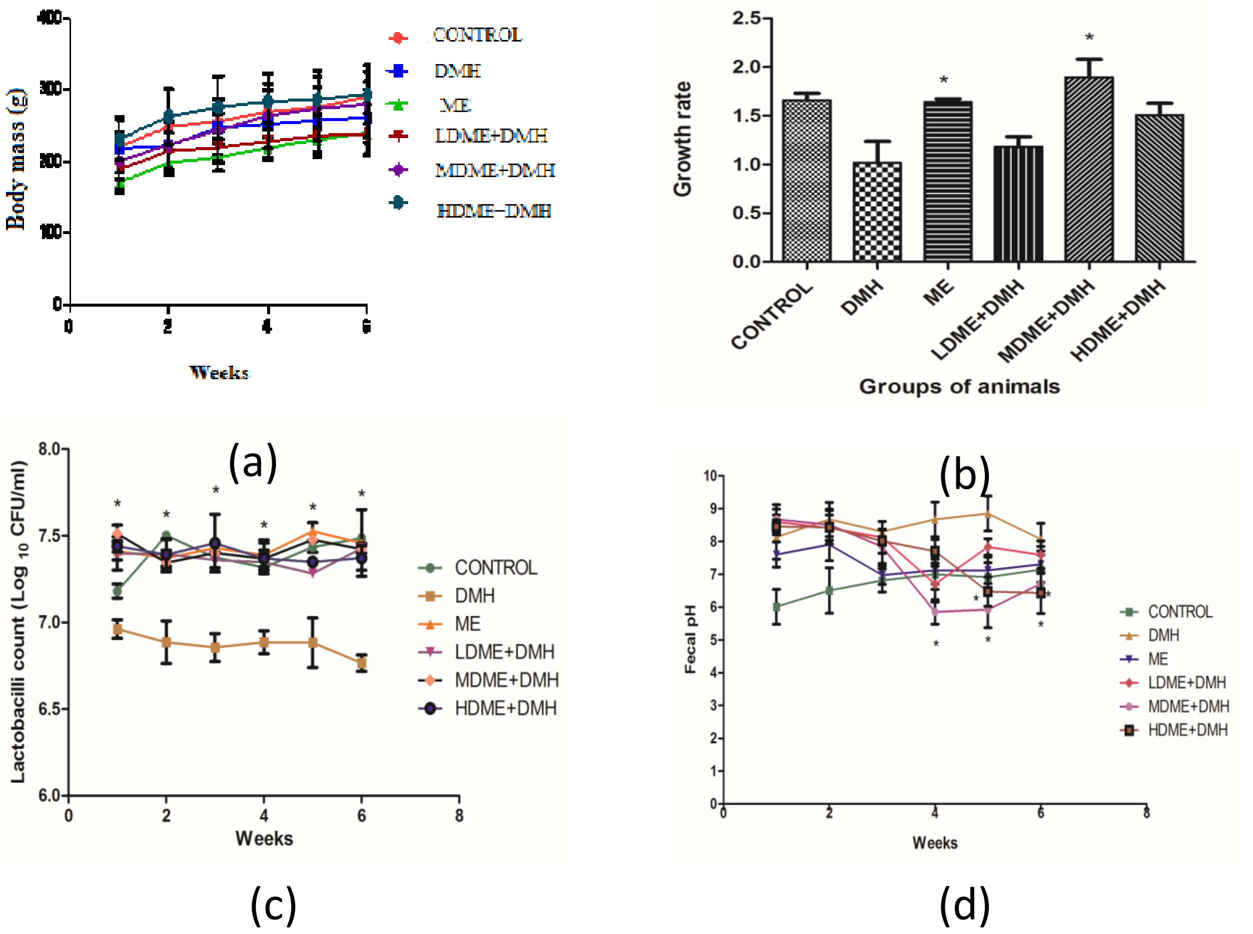

Supplementary Table 1: Primers sequences and physical parameters for various genes (β-actin, Cox-2, β-catenin, NF-**κ**B, K-ras, p53) analyzed by qRT-PCR

Gene	Primer Sequences 5' → 3'	Annealing temperature	Tm	Product size (bp)
**β- actin**	**Forward primer;** TCACCCACACTGTGCCCATCTATGA **Reverse primer;** GTCACGCACGATTTCCCTCTCAGC	60ºC	64.60 60.10	180
**Cox-2**	**Forwardprimer;** ACAGGAGAGAAGAAATGGCTGCAGAGT **Reverse primer;** CAGTATTGAGGAGAACAGATGGGATT	59ºC	65.1 61.70	198
**β-catenin**	**Forwardprimer;** ACAGCACCTTCAGCACTCT **Reverse primer;** RAAGTTCTTGGCTATTACGACA	52ºC	52.70 54.00	168
**NF-κB**	**Forwardprimer;** AGAGGATGTGGGGTTTCAGG **Reverse primer;** GCTGAGCATGAAGGTGGATG	60ºC	59.40 59.40	159
**K-ras**	**Forwardprimer;** TGACTGACAACTCCCTCATCAA **Reverse primer;** AGCCATTCGCTGCTACAAGA	55ºC	58.40 57.30	106
**p53**	**Forwardprimer;** TTCACAAAGCGTTCGTGCTG **Reverseprimer;** TTGCACAAAAACCGCTCCTG	56ºC	57.30 57.30	110

Supplementary Table 2: Fold change in expression of a) COX-2; b) β-catenin; c) NF-ĸB; d) Kras; e) p53 in various groups of animals after respective treatment by real time qPCR

a)

GROUPS	NORMALIZED AVERAGE Cq (ΔCq)	FOLD CHANGE IN EXPRESSION OVER CONTROL ($2^{-\Delta\Delta Cq}$)
CONTROL	5.473±0.075*	1
DMH	1.280±0.065	18.06±0.52
ME	4.52±0.242*	1.954±0.36*
LDME+DMH	7.256±0.221*	0.29±0.04*
MDME+DMH	7.966±0.257*	0.179±0.03*
HDME+DMH	7.926±0.075*	0.18±0.01*

Values are expressed as mean ± SD, *p<0.05 versus DMH-treated; $\Delta Cq = Cq_{GOI} - Cq_{HK}$; $\Delta\Delta Cq = \Delta Cq_{GOI} - \Delta Cq_{REF}$; Cq= quantification cycle; GOI= Gene of interest (COX-2); HK=Housekeeping gene (β-actin); REF= Reference sample (Control)

b)

GROUPS	NORMALIZED AVERAGE Cq (ΔCq)	FOLD CHANGE IN EXPRESSION OVER CONTROL ($2^{-\Delta\Delta Cq}$)
CONTROL	4.033±0.106*	1
DMH	0.740±0.270	9.981±1.84
ME	3.733±0.421*	1.551±0.73*
LDME+DMH	4.553±0.102*	0.70±0.04*
MDME+DMH	5.303±0.166*	0.42±0.04*
HDME+DMH	5.163±0.040*	0.46±0.01*

Values are expressed as mean ± SD, *p<0.05 versus DMH-treated; $\Delta Cq = Cq_{GOI} - Cq_{HK}$; $(\Delta\Delta Cq) = \Delta Cq_{GOI} - \Delta Cq_{REF}$; Cq= quantification cycle, GOI= Gene of interest (β-catenin), HK=Housekeeping gene (β-actin), REF= Reference sample (Control)

c)

GROUPS	NORMALIZED AVERAGE Cq (ΔCq)	FOLD CHANGE IN EXPRESSION OVER CONTROL ($2^{-\Delta\Delta Cq}$)
CONTROL	6.91±0.052*	1
DMH	0.873±0.179	221.529±27.91
ME	8.256±0.096*	0.393±0.02*
LDME+DMH	3.746±0.159*	9.02±1.0*
MDME+DMH	8.823±0.105*	0.27±0.01*
HDME+DMH	8.706±0.126*	0.29±0.03*

Values are expressed as mean ± SD, *p<0.05 versus DMH-treated; $\Delta Cq = Cq_{GOI} - Cq_{HK}$; $(\Delta\Delta Cq) = \Delta Cq_{GOI} - \Delta Cq_{REF}$; Cq= quantification cycle, GOI= Gene of interest (NF-ҜB), HK=Housekeeping gene (β-actin), REF= Reference sample (Control).

d)

GROUPS	NORMALIZED AVERAGE Cq (ΔCq)	FOLD CHANGE IN EXPRESSION OVER CONTROL ($2^{-\Delta\Delta Cq}$)
CONTROL	3.543±0.056	1
DMH	1.926±0.032	3.063±0.05
ME	3.65±2.66*	0.325±0.07*
LDME+DMH	5.103±0.246*	0.34±0.06*
MDME+DMH	5.683±0.140*	0.23±0.02*
HDME+DMH	5.346±0.092*	0.29±0.02*

Values are expressed as mean ± SD, *p<0.05 versus DMH-treated; $\Delta Cq = Cq_{GOI} - Cq_{HK}$; $(\Delta\Delta Cq) = \Delta Cq_{GOI} - \Delta Cq_{REF}$; Cq= quantification cycle, GOI= Gene of interest (K-ras), HK=Housekeeping gene (β-actin), REF= Reference sample (Control).

e)

**GROUPS**	**NORMALIZED AVERAGE Cq (ΔCq)**	**FOLD CHANGE IN EXPRESSION OVER CONTROL ($2^{-\Delta\Delta Cq}$)**
CONTROL	5.373±0.213	1
DMH	6.186±0.050	0.57±0.01
ME	6.863±0.090	0.312±0.06
LDME+DMH	5.440±0.085	0.97±0.05
MDME+DMH	3.030±0.138*	5.16±0.493*
HDME+DMH	1.323±0.190*	105.65±13.81*

Values are expressed as mean ± SD, *$p<0.05$ versus DMH-treated; $\Delta Cq = Cq_{GOI} - Cq_{HK}$; $(\Delta\Delta Cq) = \Delta Cq_{GOI} - \Delta Cq_{REF}$; Cq= quantification cycle, GOI= Gene of interest (p53), HK=Housekeeping gene (β-actin), REF= Reference sample (Control).
